# Minimally invasive techniques for lateral maxillary sinus floor elevation: small lateral window and one-stage surgery—a 2–5-year retrospective study

**DOI:** 10.1038/s41368-023-00233-4

**Published:** 2023-07-11

**Authors:** Shaojingya Gao, Yao Jiang, Yangxue Yao, Songhang Li, Xiaoxiao Cai

**Affiliations:** 1 State Key Laboratory of Oral Diseases, West China Hospital of Stomatology, Sichuan University, Chengdu, China; 2 Department of Demography, Zhou Enlai School of Government, Nankai University, Tianjin, China

**Keywords:** Fixed prosthodontics, Surgery

## Abstract

This study aimed to introduce a minimally invasive technique for maxillary sinus floor elevation using the lateral approach (lSFE) and to determine the factors that influence the stability of the grafted area in the sinus cavity. Thirty patients (30 implants) treated with lSFE using minimally invasive techniques from 2015 to 2019 were included in the study. Five aspects of the implant (central, mesial, distal, buccal, and palatal bone heights [BHs]) were measured using cone-beam computed tomography (CBCT) before implant surgery, immediately after surgery (T0), 6 months after surgery (T1), and at the last follow-up visit (T2). Patients’ characteristics were collected. A small bone window (height, (4.40 ± 0.74) mm; length, (6.26 ± 1.03) mm) was prepared. No implant failed during the follow-up period (3.67 ± 1.75) years. Three of the 30 implants exhibited perforations. Changes in BH of the five aspects of implants showed strong correlations with each other and BH decreased dramatically before second-stage surgery. Residual bone height (RBH) did not significantly influence BH changes, whereas smoking status and type of bone graft materials were the potentially influential factors. During the approximate three-year observation period, lSFE with a minimally invasive technique demonstrated high implant survival rate and limited bone reduction in grafted area. In conclusion, lSFE using minimally invasive techniques was a viable treatment option. Patients who were nonsmokers and whose sinus cavity was filled with deproteinized bovine bone mineral (DBBM) had significantly limited bone resorption in grafted area.

## Introduction

Alveolar bone resorption and maxillary sinus expansion are common phenomena following tooth loss in the maxillary posterior region. Proper implant placement in this region is frequently full of challenges owing to the limited available residual bone height (RBH).^1–3^ Maxillary sinus floor elevation with a lateral (lSFE) or transcrestal (tSFE) approach is adopted to elevate the Schneiderian membrane and create sufficient bone volume for implants.^4–6^ The clinical security and efficacy of tSFE and lSFE have already been demonstrated by plenty of studies.^7–9^ As the most widely used and conventional technique, lSFE is conducted to prepare a bone window in the lateral sinus wall and lift its membrane for placement of the bone graft materials and implants.^10^ Compared with tSFE, although lSFE provides direct intraoperative vision and unrestricted instrument operation, it is more invasive, with longer surgical duration, and more postoperative morbidity.^11,12^

In order to overcome these drawbacks, the lSFE procedures have been continuously modified. A conservative strategy with a less-invasive window design was lately put forward by a couple of researchers. It was demonstrated that lSFE with a small window was as clinically successful as that with a large window for achieving bone augmentation and implant survival.^13,14^ During lSFE surgery with a small bone window, opening the bone window and filling graft materials required a shorter duration. A shorter surgery duration and smaller flap size can lead to less edema and pain among patients.^14,15^ Visual analog scales (VAS) diagrams were utilized to analyze patients’ post-surgical discomforts every seven days after surgery, and patients with a small lateral bone window reported pain relief at 7, 14, and 30-day follow-up.^14^ Moreover, small bone windows played a critical role in managing and preventing intraoperative complications.^16^ As the most frequent intraoperative complication, membrane perforations were closely related to a larger window area.

Not only can lSFE be performed as pre-implantation surgery (two-stage surgery), but implants can also be placed at the same time (one-stage surgery), if the primary stability can be achieved. A recent systematic review revealed that the 5-year implant survival rate ranged from 88.6% to 100%, with no significant differences between one- and two-stage surgeries.^17^ Undoubtedly, compared with two-stage surgery, one-stage surgery can be treated as a less invasive, time-saving, and cost-effective clinical option. Thus, a novel minimally invasive technique for lSFE that combined small bone access with simultaneous implant placement was proposed.

One possible focus of maxillary sinus floor elevation was the long-term stability of the bone graft.^18^ Autogenous bone is generally considered the gold standard graft material due to its superb osteoinductive, osteoconductive, and osteogenic features.^19^ However, a major concern is that autogenous bone grafts required donor site surgery and had a high and unpredictable resorption rate.^20–22^ To overcome the drawbacks, different forms of biomaterials were proposed, including allogenic, xenogeneic, and synthetic bones. In particular, deproteinized bovine bone mineral (DBBM) is likely to be one of the most promising candidates, owing to its slow substitution rate, superior space maintenance capability, and high osteoconductive properties.^23–25^ A study demonstrated that a composite of autogenous bone and DBBM achieved clinical success in peri-implant bone augmentation.^26–28^ To assess the peri-implant bone augmentation, various imaging approaches were utilized.^29^ Lately, cone-beam computed tomography (CBCT) was considered as a promising three-dimensional (3D) option in evaluating the extent of peri-implant bone augmentation surrounding implants.^14,23,27,30^ However, 3D analysis of the grafted area of lSFE with a small window was scant and had short follow-up periods.^14,30,31^ In the consistency of these studies, authors verified that the stability of implants and excellent osteogenic capacities were detected six months after small antrostomy surgery.

The primary objective of our retrospective study was to meticulously introduce a minimally invasive technique for lSFE in terms of minimal lateral bone access and simultaneous implant placement. The long-term stability of the bone graft area was analyzed by circumferentially evaluating the peri-implant bone heights (BH) from CBCT images, furthermore, the potential influencing factors related to bone resorption in the maxillary sinus were investigated.

## Results

### Patient characteristics

**Fig. 1:**
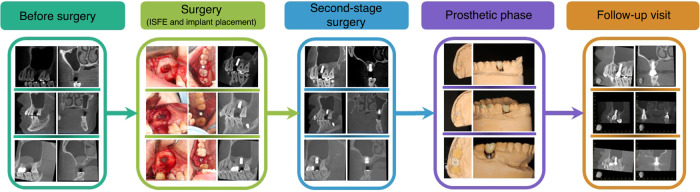
Schematic diagram of the retrospective study

**Fig. 2:**
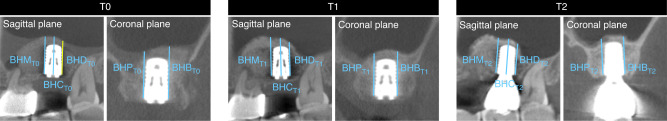
Radiographic assessment. Lower end of the line represents implant platform, upper end of the line represents uppermost level of the grafted sinus floor. T0: immediately after implant surgery; T1: before second-stage surgery; T2: at the last follow-up visit. BH_T0_, BH_T1_, BH_T2_: BH was measured at T0, T1 and T2 respectively. BH was measured at five aspects of each implant: central (BHC), mesial (BHM), and distal (BHD), buccal (BHB), and palatal (BHP) aspects

The schematic diagram of the retrospective study was shown in Fig. 1, and schematic diagram of radiographic assessment was shown in Fig. 2. The duration for all the patients from T1 to T0 was 6 months. The mean follow-up period was 44.36 months (SD = 21.31, ranging from 17 to 72). No significant complications were detected during the follow-up. The participants’ characteristics were presented in Table 1. Thirty participants (8 males and 22 females), the average age of 45.13 years (SD = 15.49, ranging from 18 to 70) who underwent maxillary sinus floor elevation using a minimally invasive technique were included in the study. Among the participants, five were smokers and 25 were non-smokers. 11 participants had a history of periodontitis, and 19 did not. One participant was placed at the first premolar; five participants were placed at the second premolar; 24 participants were placed at the first molar, and three participants were placed at the second molar. The participants’ mean RBH was 3.39 mm (SD = 1.30, ranging from 1.13 to 6.24). All Straumann implants were cylinder, while Dentium and Nobel implants were tapered. The vast majority of participants had Straumann implants with an insertion torque of 35 N·cm. The mean length in mm of the implant protruding into the sinus cavity (LIPSC) was 5.41 (SD = 1.43) and ranged from 1.66 to 8.50. Perforations of the Schneiderian membrane occurred in three patients (incidence, 10%). All the perforations were small and covered with an absorbable collagen membrane during surgery. No special treatment was conducted except regular postoperative routine medication. During the overall follow-up period, none of the implants failed.

**Table 1:** Characteristics of patients who underwent minimally invasive techniques for lateral maxillary sinus floor elevation

Characteristics	
Male/Female	8/22
Mean age (years old)	45.13 (15.49)
Smokers/non-smokers	5/25
Number of patients with periodontitis/without periodontitis	11/19
Number of patients with sinus membrane thickness of 1–2 mm/less than 1 mm	11/19
Implant site of first premolar/second premolar/first molar/second molar	1/2/24/3
Mean RBH	3.39 (1.30)
Implant system of Straumann/Dentium/Nobel	27/2/1
Implant diameter of 3.3 mm/4.1 mm/≥4.8 mm	2/7/21
Number of patients with implant length of 8 mm/10 mm	18/12
Initial stability of 15 N·cm/20 N·cm/25 N·cm/30 N·cm/35 N·cm	2/2/6/4/16
Number of patients with bone meal brand of DBBM/β-TCP	22/8
Amount of bone meal of 0.25 g/0.50 g/0.75 g	8/19/3
Number of patients with collagen membrane/without collagen membrane	23/7
Mean LIPSC	5.41(1.43)
Number of patients with membrane perforation/without membrane perforation	3/27
Number of implants placed/lost	30/0

*RBH* residual bone height, *LIPSC* length of the implant protruding into the sinus cavity, *β-TCP* β-tricalcium phosphate

**Fig. 3:**
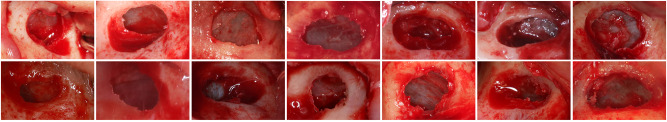
Representative images of the small lateral bone window prepared in our study

**Fig. 4:**
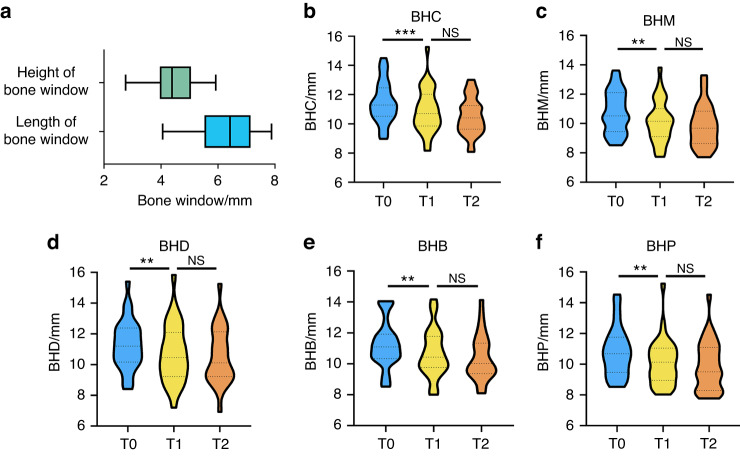
Bone window dimensions and bone height of five aspects of implants. **a** Bone window dimensions. **b**–**f** Bone height and analysis at T0, T1 and T2. *** *P* < 0.01, ** *P* < 0.05. T0, immediately after bone grafting; T1, before second-stage surgery; T2, last follow-up visit. BH, distance between implant platform and uppermost level of bone graft within sinus cavity. BH was measured at five aspects of each implant: central (BHC), mesial (BHM), and distal (BHD), buccal (BHB), and palatal (BHP) aspects

A small rectangular lateral bone access was prepared to elevate the Schneiderian membrane (Fig. 3). The dimensions of lateral bone windows prepared in the study were (4.40±0.74) mm in height and (6.26 ± 1.03) mm in length (Fig. 4a). The average surgery time was 45.9 min (SD = 6.33, ranging from 41.0 to 50.8).

### Radiation analysis

All patients in the study were examined with radiation analysis of CBCT at T0 (immediately after implant surgery), T1 (before the second-stage surgery), and T2 (at the last follow-up visit). BH was defined as the distance between the implant platform and the uppermost level of bone graft in sinus cavity. BH was measured at the five aspects of each implant: central (BHC), mesial (BHM), distal (BHD), buccal (BHB), and palatal (BHP) aspects (Fig. 2). To analyze the stability of the grafted area in sinus cavity, BH changes from T0 to T1 (ΔBH_T0–T1_) and from T0 to T2 (ΔBH_T0–T2_) were performed.

As shown in Fig. 4 and Table 2, from T0 to T1, all changes in BH showed a significant difference, whereas from T1 to T2, the BH was relatively stable. Postoperatively, the vertical bone gain was 8.07 mm (SD = 1.63, ranging from 5.56 to 11.54). The mean BHC was 11.46 ± 1.40 mm at T0, and that was (10.92 ± 1.52) mm at T1. Thus, from T0 to T1, the mean BHC decreased significantly by (0.54 ± 0.97) mm (*P* < 0.01). Moreover, ΔBHM_T0–T1_, ΔBHD_T0–T1_, ΔBHB_T0–T1_, and ΔBHP_T0–T1_ significantly decreased to (0.64 ± 1.40) mm (*P* < 0.05), (0.55 ± 1.17) mm (*P* < 0.05), (0.56 ± 1.32) mm (*P* < 0.05), and (0.70 ± 0.92) mm (*P* < 0.01), respectively.

**Table 2:** Mean, standard deviation, and *P* values of bone height changes (mm)

Outcome	T0	T1	ΔBH_T0–T1_	T0–T1 (*P* value)
BHC	11.46 (1.40)	10.92 (1.52)	−0.54 (0.97)	0.00***
BHM	10.78 (1.43)	10.14 (1.47)	−0.64 (1.40)	0.01**
BHD	11.30 (1.61)	10.75 (1.92)	−0.55 (1.17)	0.01**
BHB	11.25 (1.52)	10.70 (1.57)	−0.56 (1.32)	0.03**
BHP	10.81 (1.60)	10.12 (1.51)	−0.70 (0.92)	0.00***

***P < 0.01, **P < 0.05. Standard deviations are in parentheses; T0, immediately after bone grafting; T1, 6 months after implant surgery; ΔBH_T0–T1_ represents the bone height changes from T0 to T1

### Statistical analysis

The results of the Pearson correlation analysis showed that all the correlating coefficients of the BH changes were positive and statistically significant, indicating significant correlations among ΔBHC_T0–T1_, ΔBHM_T0–T1_, ΔBHD_T0–T1_, ΔBHB_T0–T1_, and ΔBHP_T0–T1_ (Table 3).

**Table 3:** Pearson correlation analysis of ΔBHC_T0–T1_, ΔBHM_T0–T1_, ΔBHD_T0–T1_, ΔBHB_T0–T1_, and ΔBHP_T0–T1_

Outcome	ΔBHC_T0–T1_	ΔBHM_T0–T1_	ΔBHD_T0–T1_	ΔBHB_T0–T1_	ΔBHP_T0–T1_
ΔBHC_T0–T1_	1.00				
ΔBHM_T0–T1_	0.81***	1.00			
	(0.00)				
ΔBHD_T0–T1_	0.78***	0.530***	1.00		
	(0.00)	(0.00)			
ΔBHB_T0–T1_	0.86***	0.78***	0.70***	1.00	
	(0.00)	(0.00)	(0.00)		
ΔBHP_T0–T1_	0.63***	0.60***	0.54***	0.70***	1.00
	(0.00)	(0.00)	(0.00)	(0.00)	

*** *P* < 0.01; *P* values are in parentheses

**Fig. 5:**
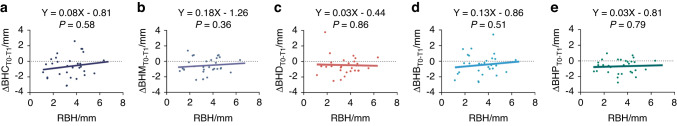
The scatter plots with fitted value lines of the relationship between RBH and ΔBHT0–T1. **a**–**e** Linear regression line of RBH and ΔBHC_T0–T1_, ΔBHM_T0–T1_, ΔBHD_T0–T1_, ΔBHB_T0–T1_, and ΔBHP_T0–T1_, respectively. ΔBH_T0–T1_ represents the bone height changes from T0–T1. RBH represents residual bone height

Scatter plots with fitted value lines (Fig. 5) showed a linear relationship between pre-surgery RBH and ΔBHC_T0–T1_, ΔBHM_T0–T1_, ΔBHD_T0–T1_, ΔBHB_T0–T1_, and ΔBHP_T0–T1_. As Fig. 5 displayed, the trends of fitting value lines were relatively smooth, which indicated that no correlations were detected between the ΔBH_T0–T1_ of the five aspects and RBH (*P* > 0.05).

BH changes were compared among different groups according to patients’ characteristics. The ΔBHC_T0–T1_, ΔBHM_T0–T1_, ΔBHB_T0–T1_, and ΔBHP_T0–T1_ of the smoker group were significantly greater than those of the non-smoker group. Compared with the group that used DBBM, the β-tricalcium phosphate (β-TCP) group showed significantly greater changes in ΔBHC_T0–T1_, ΔBHM_T0–T1_, ΔBHD_T0–T1_, ΔBHB_T0–T1_, and ΔBHP_T0–T1_ (Table 4).

**Table 4:** Comparison of mean ΔBH according to the characteristics of patients

Characteristics	Mean bone height change (mm)				
	ΔBHC_T0–T1_	ΔBHM_T0–T1_	ΔBHD_T0–T1_	ΔBHB_T0–T1_	ΔBHP_T0–T1_
Gender					
Male	−0.26 (0.76)	0.04 (1.51)	−0.38 (0.78)	0.20 (1.41)	−0.29 (0.86)
Female	−0.65 (1.03)	−0.88 (1.31)	−0.61 (1.30)	−0.83 (1.21)	−0.85 (0.91)
*P* value	0.34	0.11	0.64	0.06*	0.15
Smoking					
Smoker	−1.22 (0.66)	−1.86 (1.31)	−1.30 (0.65)	−1.58 (1.11)	−1.80 (0.87)
Non-smoker	−0.41 (0.97)	−0.39 (1.30)	−0.40 (1.20)	−0.35 (1.28)	−0.48 (0.77)
*P* value	0.09*	0.03**	0.12	0.06*	0.00***
Periodontitis					
With periodontitis	−0.58 (1.00)	−0.83 (1.49)	−0.64 (0.71)	−0.62 (1.75)	−0.69 (1.04)
Without periodontitis	−0.52 (0.98)	−0.53 (1.37)	−0.50 (1.39)	−0.52 (1.05)	−0.70 (0.87)
*P* value	0.86	0.58	0.75	0.84	0.96
Sinus membrane thickness					
Less than 1 mm	−0.59 (0.98)	−0.83 (1.33)	−0.38 (1.32)	−0.48 (1.41)	−0.72 (0.98)
1–2 mm	−0.46 (0.99)	−0.30 (1.52)	−0.84 (0.84)	−0.68 (1.21)	−0.66 (0.84)
*P* value	0.72	0.32	0.31	0.70	0.88
Residual bone height					
<4 mm	−0.70 (1.05)	−0.82 (1.47)	−0.60 (1.41)	−0.83 (1.24)	−0.78 (0.95)
≥4 mm	−0.31 (0.81)	−0.36 (1.30)	−0.47 (0.74)	−0.14 (1.38)	−0.58 (0.89)
*P* value	0.29	0.39	0.76	0.17	0.57
Implant length					
8 mm	−0.46 (0.99)	−0.48 (1.20)	−0.45 (1.38)	−0.65 (1.14)	−0.60 (0.84)
10 mm	−0.66 (0.96)	−0.87 (1.69)	−0.70 (0.81)	−0.42 (1.60)	−0.85 (1.04)
*P* value	0.59	0.47	0.58	0.65	0.48
Type of bone graft material					
DBBM	−0.22 (0.83)	−0.17 (1.23)	−0.30 (1.13)	−0.20 (1.28)	−0.53 (0.86)
β-TCP	−1.43 (0.77)	−1.92 (1.00)	−1.24 (1.06)	−1.53 (0.91)	−1.17 (0.97)
*P* value	0.00***	0.00***	0.05**	0.01**	0.09*
Collagen membrane					
With collagen membrane	−0.63 (0.98)	−0.64 (1.46)	−0.75 (0.94)	−0.60 (1.41)	−0.88 (0.97)
Without collagen membrane	−0.26 (0.95)	−0.63 (1.28)	0.11 (1.65)	−0.40 (1.03)	−0.10 (0.29)
*P* value	0.40	0.99	0.09*	0.74	0.05**
Membrane perforation					
With membrane perforation	−0.98 (0.07)	−0.42 (0.15)	−0.64 (0.24)	−0.96 (0.66)	−1.11 (0.68)
Without membrane perforation	−0.49 (1.01)	−0.66 (1.47)	−0.54 (1.24)	−0.51 (1.38)	−0.65 (0.94)
*P* value	0.42	0.78	0.89	0.58	0.43

*RBH* residual bone height, *LIPSC* length of the implant protruding into the sinus cavity, *β-TCP* β-tricalcium phosphate

*P < 0.1; Standard deviations are in parentheses. ***P < 0.01, **P < 0.05, *P < 0.1

Considering the effects of multiple factors on BH changes, the linear mixed models were employed. The results in Table 5 estimated by the linear mixed models suggested that smoking had a higher possibility of decreasing BH, which was significantly different among ΔBHC_T0–T1_ (*P* < 0.1), ΔBHB_T0–T1_ (*P* < 0.1), and ΔBHP_T0–T1_. (*P* < 0.1). However, the effects of smoking on ΔBHM_T0–T1_ and ΔBHD_T0–T1_ were insignificant. Furthermore, the filling sinus with DBBM was significantly associated with ΔBHC_T0–T1_ (*P* < 0.1) and ΔBHB_T0–T1_ (*P* < 0.1). Overall, except for smoking and bone graft materials, no other factors were found to be significantly correlated with changes in BH.

**Table 5:** Linear mixed models for analyzing factors influencing ΔBH

Variables	ΔBHC_T0–T1_	ΔBHM_T0–T1_	ΔBHD_T0–T1_	ΔBHB_T0–T1_	ΔBHP_T0–T1_
Female as reference					
Male	−0.61	0.59	−0.96	−0.55	0.62
	(0.45)	(0.91)	(0.76)	(0.54)	(0.60)
Age	−0.03	−0.03	−0.03	−0.02	−0.03
	(0.02)	(0.04)	(0.04)	(0.03)	(0.03)
Non-smoker as reference					
Smoking	−1.11*	−1.43	−0.98	−1.44*	−1.49*
	(0.51)	(1.03)	(0.87)	(0.61)	(0.67)
Without periodontitis as reference					
With periodontitis	−0.46	−1.67	−0.47	−0.77	−0.47
	(0.49)	(0.99)	(0.83)	(0.58)	(0.65)
Sinus membrane thickness of 1–2 mm as reference					
Sinus membrane thickness less than 1 mm	0.18	−0.23	0.55	0.48	−0.87
	(0.49)	(0.99)	(0.83)	(0.58)	(0.64)
Implanting site of first premolar as reference					
Second premolar	0.78	0.00	1.81	−2.40	1.85
	(1.24)	(2.51)	(2.10)	(1.48)	(1.64)
First molar	1.54	0.30	1.82	−1.33	0.94
	(1.12)	(2.26)	(1.89)	(1.33)	(1.47)
Second molar	0.98	0.88	1.01	−1.47	−0.62
	(1.27)	(2.56)	(2.14)	(1.51)	(1.67)
RBH	0.30	0.81	−0.25	0.69	−0.13
	(0.33)	(0.67)	(0.56)	(0.40)	(0.44)
Straumann implant as reference					
Dentium	2.13	2.16	3.27	3.00*	0.85
	(1.21)	(2.44)	(2.05)	(1.44)	(1.60)
Nobel	1.18	−1.27	2.40	0.52	2.14
	(1.50)	(3.02)	(2.53)	(1.79)	(1.97)
Implant diameter of 3.3 mm as reference					
4.1 mm	2.17*	3.00	1.61	1.98	1.45
	(0.90)	(1.81)	(1.52)	(1.07)	(1.18)
4.8 mm	1.62	2.57	0.46	1.93	0.51
	(1.25)	(2.52)	(2.11)	(1.49)	(1.64)
Initial stability of 15 N·cm as reference					
20 N·cm	−2.12	−0.04	−1.34	−1.34	−1.43
	(1.09)	(2.21)	(1.85)	(1.31)	(1.44)
25 N·cm	0.63	1.10	1.44	1.30	0.81
	(0.80)	(1.61)	(1.35)	(0.95)	(1.05)
30 N·cm	−1.28	0.04	−1.41	−0.62	−0.79
	(0.89)	(1.80)	(1.51)	(1.06)	(1.18)
35 N·cm	−1.42	0.76	−1.46	−0.28	−0.90
	(0.84)	(1.69)	(1.42)	(0.10)	(1.10)
Bone filling material of β-TCP as reference					
DBBM	1.33*	1.82	0.50	1.46*	0.23
	(0.59)	(1.18)	(0.99)	(0.70)	(0.77)
Bone graft of 0.25 g as reference					
0.5 g	0.50	0.43	0.93	0.78	0.06
	(0.57)	(1.15)	(0.96)	(0.68)	(0.75)
0.75 g	1.37	−0.37	1.57	1.27	0.15
	(0.97)	(1.95)	(1.63)	(1.15)	(1.27)
Without collagen membrane as reference					
With collagen membrane	0.21	0.07	−0.10	0.46	−1.24
	(0.52)	(1.06)	(0.89)	(0.62)	(0.69)
Without membrane perforation as reference					
With membrane perforation	0.67	0.82	0.84	0.70	0.32
	(0.62)	(1.25)	(1.04)	(0.74)	(0.81)
LIPSC	−0.24	0.15	−0.66	−0.04	−0.26
	(0.32)	(0.64)	(0.54)	(0.38)	(0.42)
Constant	−2.50	−7.13	2.43	−3.99	2.83
	(4.51)	(9.09)	(7.62)	(5.37)	(5.94)
R-squared	0.92	0.85	0.85	0.94	0.85

*RBH* residual bone height, *LIPSC* length of the implant protruding into the sinus cavity, *β-TCP* β-tricalcium phosphate, *LIPSC* length of the implant protruding into the sinus cavity

*P < 0.1; Standard errors are in parentheses

Bold values indicates statistical significant P values

## Discussion

The primary objective of this study was to describe a novel minimally invasive technique featuring a small bone window access and one-stage surgery for lSFE. Implant survival rate, long-term stability of the grafted area, and potentially influential factors were investigated. The retrospective study demonstrated that the minimally invasive technique was a reliable clinical procedure with a 100% implant survival rate during the entire follow-up period. The 3D stability of the bone graft in maxillary sinus was detected, and the height of bone graft decreased rapidly during the first 6 months. Smoking and the type of bone graft materials were significant explanatory variables for BH changes. There was no statistically significant correlation between BH changes and preoperative RBH.

The lSFE technique was first introduced by Boyne and James using a large round burr to open a window at the lateral bone.^32^ The size of the bone window remains a controversial issue. A wide flap and a large bone window were proposed to allow maximum accessibility and sufficient visualization of the surgical area.^33^ The surface area of a large window was generally larger than 80 mm^2^. (10 mm in length and 8 mm in height), fabricated by piezoelectric devices or round burrs.^34–36^ However, as a significant source of blood supply contributing to bone formation, the lateral bone was destroyed by a large bone window to a great extent. A number of studies have demonstrated that a large lateral window negatively influenced vascularization and bone formation in the grafted area.^30,37,38^ A recent study by Zhu et al. reached a similar conclusion that patients with a small bone window exhibited increased osteogenic potential, including higher mineral apposition rate, higher bone formation rate, and larger new bone area.^39^ The possible concerns of the small bone window were restricted visibility and limited access to lift the Schneiderian membrane and fill the bone graft. A study by Baldini and colleagues dispelled concerns.^14^ Compared with large window group, preparing a small bone window and performing sinus filling took a shorter time. Membrane elevation in the small window group could be performed as quickly as in the large-window group. This indicated that a small bone window could provide surgeons with adequate accessibility and visualization. According to the studies providing data on the dimensions of a small bone window, a length of ~6–8 mm and a height of 4–6 mm were reported.^14,16,30,39–41^ Generally, the surface area of a small lateral bone window was less than 40 mm^2^. The area of the small bone window prepared in our study was ~27 mm^2^,which was consistent with the results of the previous studies.

Perforations of the Schneiderian membrane are the most frequent intraoperative complication of lSFE. The reported incidence rate ranged from 10% to 60%.^42^ Of 30 patients, three suffered from membrane perforations, in accordance with the literature. Al-Dajani and colleagues systematically reviewed the incidence of membrane perforations in patients with lSFE. In this review, 12 studies and 388 membrane perforations were included.^43^ The incidence of membrane perforations ranged from 3.6% to 41.8%, leading to a weighted prevalence of 23.5%. Whether membrane perforations influenced implant survival remained under discussion. Hernandez-Alfaro and colleagues verified that membrane perforation size had a negative effect on implant survival rate.^44^ When the perforation size was larger than 5 mm, bioabsorbable membranes were utilized to repair the perforations, probably resulting in decreased bone formation and implant failure.^45^ However, with the development of surgical equipment and techniques, large membrane perforations do not usually occur. An up to 20-year retrospective study showed that membrane perforations were unlikely to influence implant survival when membrane perforations were treated properly and carefully.^46^

Initial RBH is considered a predictive indicator for the clinical option of one- and two-stage surgeries for maxillary sinus elevation. When the RBH is 5 mm or less, the lSFE is usually preferred. In particular, two-stage surgery is recommended in cases with RBH < 4 mm.^47,48^ With innovations in surgical equipment and technology, the indications for lSFE with one-stage surgery have expanded. lSFE with one-stage surgery is treated as a feasible and reliable clinical option, even with RBH < 4 mm. A previous animal study was conducted to provide evidence that implant sites with 2 mm RBH showed similar stability as implants with 8 mm RBH at the time of implant placement.^49^ Stacchi et al. in a histomorphometric study pointed out that a sufficient degree of newly formed bone tissue could be recognized regardless of RBH.^50^ According to the observation at the 6-month follow-up, our study also verified that implant survival rate was not significantly influenced by RBH. A comparative evaluation was conducted to demonstrate that simultaneous implant placement could be accomplished at the site with RBH < 5 mm. During the 5-year observation period, the survival rate did not show significant differences between the two RBH groups (<5 mm and >5 mm).^51^ Peleg et al. suggested that under the premise of meticulous surgical planning and skills, implants could be simultaneously placed in sites with at least 1 mm of RBH, resulting in an extraordinarily high survival rate within an observation period of 9 years.^52^ A long-term retrospective research by Han and the colleagues investigated the 10 and 20-year cumulative survival rates of implants placed simultaneously with lSFE, and they did not observe significant differences in the survival rates of implants placed in different RBH during the 10-year period. The survival rate was markedly lower for implants placed in <3 mm RBH than for those placed in ≥3 mm RBH at 20 years.^46^ Yet it is worth noting that the 20-year survival rate for implants placed in <3 mm RBH was 78.8%, which was considered acceptable by the researchers.

The strong correlation between smoking status and BH changes in lSFE performed using a minimally invasive technique was demonstrated in the present study. Currently, several studies are available to confirm this finding. Schwartz-Arad et al. suggested that peri-implant BH showed greater resorption in smokers.^53^ Guan and the colleagues in a clinical retrospective study showed that smokers had a higher bone loss of 0.7 mm compared with non-smokers.^54^ This study adopted a linear mixed model to describe smoking status as a potential influencing factor associated with bone graft resorption in the sinus cavity. More air pressure was placed in the maxillary sinus of smokers, leading to great resorption of the bone graft and a significant decrease in its height. The peri-implant microbiome, related to osseointegration, can also be affected by nicotine.^55^ These were possible reasons why smoking status was the key factor in influencing bone graft resorption. However, based on the retrospective radiographic research carried out by Geurs et al., although a greater change in graft height was found in the smoking group at the 3-year follow-up, there was no statistical significance between the smoking and nonsmoking groups.^56^ Trombelli et al. also confirmed that smoking had a limited impact on radiographic outcomes 6 months after maxillary sinus elevation.^57^ These two studies performed analysis of variance (ANOVA) and U-test and found no significant differences in mean graft changes between smokers and nonsmokers.^56,57^ However, the above results ignored the other possible factors that may influence the mean graft changes, such as sex, age, and especially the type of bone graft materials, which might produce estimation errors to a large extent. In this study, we considered the factors that may affect the mean graft changes as much as possible and used mixed linear regression to explore the possible risk factors for graft height changes.

Autogenous bone results in excellent bone formation, but it may be associated with postoperative morbidity, limited quantities, and unpredictable resorption rate. As one of the most clinically and histologically investigated graft materials, DBBM was safe and effective for sinus lift procedures, especially lSFE. The previous studies demonstrated slow degradation rate and effective bone regeneration of DBBM indicating that it was a promising candidate in lSFE procedures.^23,27,58^ Composite of autogenous bone and DBBM provided the advantages of autogenous bone while restricting its undesired effects. Compared with autogenous bone, β-TCP graft materials also exhibited lower resorption speed. β-TCP might be gradually resorbed and replaced by newly formed bone before the second-stage surgery.^59^ β-TCP graft materials were investigated to provide space maintenance leading to high and long-term implant survival rates. Trombelli et al. reported when DBBM or β-TCP was used to fill sinus cavity in SFE. A significant remodeling was observed in the β-TCP group from immediate post-surgery to 6 months after surgery.^60^ Histomorphometry studies showed that DBBM exhibited a greater ratio of newly formed bone compared to β-TCP before the second-stage surgery.^61,62^ Overall, these studies seemed to agree with the results in our study. When autogenous bone combined with DBBM was used in lSFE with a minimally invasive technique, limited bone resorption in sinus cavity was detected.

In order to exactly understand the results of the present study, the limitations are as follows. First, it is a retrospective study with a small sample size, which probably caused inherent bias in the results. There was no control group to compare patients with lSFE using a large bone window or tSFE. Thus, a prospective study or randomized controlled study with a larger sample size is preferred. Second, although a 3D analysis device (CBCT) was employed to determine the stability of the grafted area, the volume of bone gain was not directly analyzed. Instead of volumetric analysis, the peri-implant BH was measured circumferentially; notwithstanding, previous studies have suggested that this is a reliable option for measuring bone gain in the sinus cavity.^30,54,58^ Third, the 2-year observation period was relatively short, despite this being the longest follow-up period in the study of lSFE with a small bone window. Further clinical trials with longer follow-up periods are warranted.

## Materials and methods

Thirty patients, who underwent lSFE with minimally invasive technique from May 2015 to November 2019 at West China Hospital of Stomatology, Sichuan University were included in the study. The study was followed strengthening the reporting of the observational studies in epidemiology (STROBE) guidelines. The retrospective study was performed to fully conform to the World Medical Association Declaration of Helsinki.^63^ Ethics Committee of West China Hospital of Stomatology, Sichuan University approved the research procedures (WCHSIRB-CT-2022-452). All patients were informed of the study procedures and signed the informed consent.

### Inclusion criteria

1. Patients older than 18 years old.
2. Patients who signed informed consent.
3. Patients who underwent lSFE with minimally invasive technique (small lateral window and simultaneous placement of single implant).
4. Patients in good health without contraindications to implant surgery.

### Exclusion criteria

1. Pregnant and lactating patients.
2. Patients with active maxillary acute sinusitis or diseases affecting wound healing and osteogenesis.
3. Patients taking immunosuppressive drugs.
4. Patients with a history of neck or head radiotherapy.
5. Patients with bruxism.
6. Patients with a cyst in sinus.

### Features of patients with minimally invasive technique of lSFE

The features of patients were collected including (a) sex, (b) age, (c) smoking status, (d) history of periodontitis, (e) sinus membrane thickness, (f) implant sites, (g) pre-surgery RBH, (h) implant system, (i) implant diameter, (j) implant length, (k) initial stability of implants, (l) type of bone graft materials, (m) quantity of bone graft, (n) presence of collagen membrane, (o) LIPSC, (p) presence of membrane perforations, and (q) the number of lost implants.

### Surgery and prosthetic phase

**Fig. 6:**
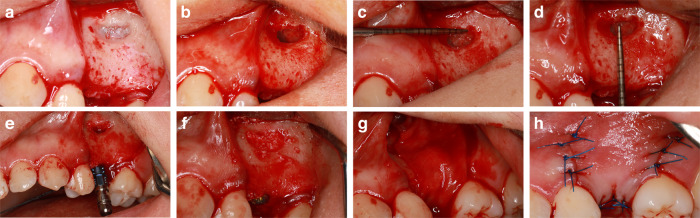
Surgery procedures of lSFE with minimally invasive technique and implant placement. **a** After raising full-thickness flap, small lateral bone window was prepared. **b** The Schneiderian membrane was then gently elevated. **c**, **d** The dimensions of bone window were measured. **e** An implant was placed immediately. **f** The space between sinus floor and sinus membrane were grafted with bone graft materials. **g** Absorbable collagen membrane was used to cover bone window and implant. **h** Mucosal flaps were carefully sutured

All surgical procedures were conducted by the same experienced surgeon (X.C., Fig. 6). At the beginning of surgery, local anesthesia (primacaine) was administered to the maxillary posterior area. Following crestal and vertical incisions, the mucoperiosteal flap was fully raised to explore the lateral bone of the maxillary sinus. A small rectangular lateral bone window was opened, and the Schneiderian membrane was carefully detached from the sinus floor using a DASK kit (Dentium, Seoul, South Korea). The height and length of the bone windows were measured using a periodontal probe. Simultaneously, the implants were placed. The initial stability of the implants was guaranteed in all cases. After implantation, the space between the Schneiderian membrane and the sinus floor was filled with a mixture of autogenous bone and DBBM (Bio-Oss, Geistlich Pharma, Wolhusen, Switzerland) or β-TCP (RTR, Haibo Han, China). A resorbable collagen membrane (Bio-Gide, Geistlich Pharma, Switzerland) was utilized to cover the bone windows and implant sites. Mucosal flaps were sutured with 5-0 non-absorbable polypropylene sutures (Prolene, Johnson & Johnson, USA).

Postoperatively, amoxicillin and metronidazole were prescribed three times daily for 7 days. All patients were asked to use a chlorhexidine mouthwash three times a day for 2 weeks. All sutures were removed within 10–14 days after surgery.

Six months after surgery, second-stage surgery was performed to replace the closure caps with healing abutments. The final prostheses of the single-ceramic crowns were fabricated. Patients underwent follow-up assessment every 6 months.

### Radiation analysis

All patients in the study were examined with radiation analysis of CBCT (of slice thickness, 0.25 mm) before implant surgery, immediately after implant surgery (T0), 6 months after implant surgery (before the second-stage surgery, T1), and at the last follow-up visit (T2). RBH was measured using CBCT images before implant surgery. The distance between the implant platform and the uppermost level of bone graft was defined as BH. BH was measured at five aspects of each implant at T0, T1, and T2: central (BHC), mesial (BHM), distal (BHD), buccal (BHB), and palatal (BHP) aspects. BHC measurements were made along the central axis of the implant, and BHM, BHD, BHB, and BHP analyses were performed along the axis and tangential to each side of the implant, respectively.^26,30^ Radiographic bone gain was calculated by subtracting BHC_T0_ and RBH. To analyze the stability of grafted area in the sinus cavity, BH changes from T0 to T1 (ΔBH_T0–T1_) and from T0 to T2 (ΔBH_T0–T2_) were used.

### Statistical analysis

All data analyses were performed using Stata software (StataCorp, College Station, TX, USA). All measurement variables were shown as mean ± standard deviation (SD). Significant differences in BH at T0, T1, and T2 were assessed by one-way repeated-measures ANOVA. Pearson correlation analysis was included in the present study to determine any relationships among ΔBHC_T0–T1_, ΔBHM_T0–T1_, ΔBHD_T0–T1_, ΔBHB_T0–T1_, and ΔBHP_T0–T1_. The comparisons of mean BH changes based on patients’ characteristics were analyzed using a paired *t*-test. Multivariate linear regression analysis was used to determine the possible relationship between RBH and ΔBH. A linear mixed model was adopted to determine the risk factors for ΔBH. *P* values < 0.1 were considered statistically significant.

## Conclusion

lSFE with a small lateral bone window and one-stage surgery has a 100% implant survival rate, limited bone graft resorption, and few influential factors during the approximate 5-year observation period. The present study revealed a strong negative correlation between changes in BH and smoking status, and no influence of RBH was detected. Autogenous bone mixed with DBBM was again demonstrated as one of the most promising candidates for bone graft filling of the sinus cavity.

## Data Availability

Data available within the article or its supplementary materials.
